# Role of SST, CORT and ghrelin and its receptors at the endocrine pancreas

**DOI:** 10.3389/fendo.2012.00114

**Published:** 2012-09-18

**Authors:** Chanclón Beléen, Antonio J. Martínez-Fuentes, Francisco Gracia-Navarro

**Affiliations:** ^1^Department of Cell Biology, Physiology and Immunology, University of CórdobaCórdoba, Spain; ^2^Instituto Maimónides de Investigación Biomédica de CórdobaCórdoba, Spain; ^3^Centro de Investigación Biomédica en Red Fisiopatología de la Obesidad y NutriciónCórdoba, Spain

**Keywords:** SST, CORT, ghrelin, insulin, islet, pancreas, endocrine

## Abstract

Somatostatin (SST), cortistatin (CORT), and its receptors (sst1–5), and ghrelin and its receptors (GHS-R) are two highly interrelated neuropeptide systems with a broad range of overlapping biological actions at central, cardiovascular, and immune levels among others. Besides their potent regulatory role on GH release, its endocrine actions are highlighted by SST/CORT and ghrelin influence on insulin secretion, glucose homeostasis, and insulin resistance. Interestingly, most components of these systems are expressed at the endocrine pancreas and are actively involved in the modulation of pancreatic islet function and, consequently influence glucose homeostasis. In addition, some of them also participate in islet survival and regeneration. Furthermore, under severe metabolic condition as well as in endocrine pathologies, their expression profile is severely deregulated. These findings suggest that SST/CORT and ghrelin systems could play a relevant role in pancreatic function under metabolic and endocrine pathologies. Accordingly, these systems have been therapeutically targeted for the prevention or amelioration of certain metabolic conditions (obesity) as well as for tumor growth inhibition and/or hormonal regulation in endocrine pathologies (neuroendocrine tumors). This review focuses on the interrelationship between SST/CORT and ghrelin systems and their role in severe metabolic conditions and some endocrine disorders.

## INTRODUCTION

The pancreas is a physiologically and biologically complex organ organized in exocrine and endocrine compartments which are modulated by a wide variety of neuronal and hormonal signals in an integrated manner. The exocrine function of the pancreas is performed by more than 90% of the whole pancreatic tissue and is essentially composed of acinar and ductal cells that respectively, synthesize and transport enzymes crucial for nutrient digestion at the gastrointestinal tract.

The endocrine function of the pancreas is in turn achieved by distinct cell types organized in major structures called Islet of Langerhans, which are scattered throughout the organ and are in close contact with the vascular environment. At least five major different endocrine cell types have been described: glucagon-secreting alpha-cells, insulin-secreting beta-cells, somatostatin (SST)-secreting delta-cells, ghrelin-producing epsilon-cells, and pancreatic polypeptide-producing cells. The distribution and proportion of endocrine cells within the pancreatic islets varies between species (Jain and Lammert, 2009). The coordinated production, release, action, and relationship of the above-mentioned pancreatic endocrine peptides determine the constitutive metabolic homeostasis within the organism. In this sense, the endocrine dysfunction of the gland as the impairment of insulin production triggers the development of type 2 diabetes mellitus (T2DM) therefore, resulting in an abnormal regulation of blood glucose concentration with ulterior significant complications. It is widely known the close relationship between type 2 diabetes and obesity (Venables and Jeukendrup, 2009). In this sense, in fact, obesity is a multi-factorial chronic metabolic condition that predispose to the development of T2DM and shares with the later a common feature: insulin resistance (Saltiel and Kahn, 2001). The increasing incidence of both metabolic disorders urges for the search of therapeutic targets in order to treat these pathologies and improve glucose homeostasis and insulin resistance, as well as body weight regulation. In this context, the action of the different ghrelin system components on glucose homeostasis, insulin resistance, and body weight regulation has been described and consequently, ghrelin system has been suggested as a potential drug target for the prevention or treatment of T2DM and obesity. In turn, some tissues are common targets of SST/cortistatin (CORT) and ghrelin (i.e., endocrine pancreas) and interestingly, these three molecules show a highly molecular parallelism (i.e., all peptides are processed from prepro-hormones that generate several biologically active peptides). In the present review, we analyze the literature relative to the modulation of endocrine pancreatic function by these two closely interrelated pleiotropic systems, SST/CORT, and ghrelin. Moreover, we also include its actions on glucose metabolism and insulin release as well as their possible pathophysiological role in metabolic disorders with increasing incidences as T2DM and obesity.

## SST/CORT PLEIOTROPIC SYSTEM

Somatostatin was originally isolated from ovine hypothalamus based on its potent inhibitory action on pituitary growth hormone (GH) secretion (Brazeau et al., 1973). At circulation, it appears in two biologically active forms consisting of 14 (SST-14) or 28 amino acid (aa) residues (SST-28), generated by differential post-translational processing from a common precursor molecule. Both isoforms are widely distributed in a number of organs and tissues, although they display a particular tissue-specific expression patterns. Thus, SST-14 is predominantly produced in the central nervous system and in several peripheral tissues, including the pancreas (Ballian et al., 2006), whereas SST-28 is mainly expressed by epithelial cells of the gastrointestinal tract mucosa. Such wide distribution of SST forms is consistent with its ever growing spectrum of biological and pathophysiological functions, mostly of inhibitory nature, such as inhibition of endocrine and exocrine secretions, neurotransmission, neuromodulation, gastrointestinal motility, immune system function, tumor cell growth, and pancreatic function (Patel, 1999).

A large amount of this wide biological capacity of SST is mediated via binding and activation of SST receptors, a family of five specific transmembrane proteins (named sst1–5) belonging to the superfamily of G proteins coupled receptors (GPCRs), and encoded by five distinct intronless genes (Gahete et al., 2010). All five isoforms recruit several downstream transduction signals upon SST binding such as adenylyl cyclase and calcium channels, which are two major players involved in SST inhibitory action on hormone release. Similarly to SST tissue distribution, ssts are present in abundant tissue locations and often, in SST tissue targets, several isoforms are simultaneously present in the same cell. In this context, it has been reported that ssts functionally interact with each other and even with other GPCRs to form homo- and/or heterodimers that activate different signaling cascades and consequently mediate multiple biological actions (Moller et al., 2003).

The pleiotropic activity featured by SST fits well with both its ample tissue distribution and its multiple receptors. Moreover, it can also be likely related to the existence of a highly similar peptide of the same family, CORT, which was originally discovered in frogs and subsequently in rodents and humans (de Lecea et al., 1996; Tostivint et al., 1996). Like SST, CORT is the product of an enzymatically processed precursor, CORT prepro-peptide, which shares high homology with SST precursor. SST and CORT precursors are encoded by two different genes that evolved from a common ancestral gene by a duplication mechanism (Tostivint et al., 1996; Gahete et al., 2010). Similar to that described for SST, processing of CORT precursor generates diverse mature peptides as CORT-14 and -29 in rodents and CORT-17 and -29 in humans. In addition, CORT- and SST-mature forms shares 11 aas, which include two cysteine residues responsible for their characteristic cyclic structure and the FWKT core (Phe7-Trp8-Lys9-Thr10), a crucial motif for receptor binding. Thus, their differences are located at the N- and C-terminal regions. In sum, CORT and SST sequence identity and structural homology explain well their close pharmacology, specifically, their comparable subnanomolar binding affinity to the five sst isoforms.

Although CORT was initially discovered in the brain and it is especially abundant in the cortex (where its name comes from), further reports showed that this neuropeptide is also widely distributed at peripheral tissues including gastrointestinal tract and pancreas. Based on its ability to activate ssts, CORT exhibits almost endocrine and most non-endocrine actions of SST (Broglio et al., 2008; Gahete et al., 2008). Actually, their main functional divergences, reside in the ability of CORT to promote sleep functions, modulate locomotor activity, exert potent anti-inflammatory actions in experimental models of inflammatory and autoimmune disorders (de Lecea et al., 1996; Gonzalez-Rey and Delgado, 2008) and, its influence on atherogenesis (Gonzalez-Rey and Delgado, 2008; Liu et al., 2010; Souza-Moreira et al., 2011).

Differential actions between SST and CORT possibly reside in the ability of CORT to interact, in addition to ssts, with the Mas-related gene 2 receptor (MrgX2), a former orphan receptor originally suggested as specific for CORT. However, this receptor appears to be a quite promiscuous GPCR that also shows some specificity for proadrenomedullin and related peptides (Nothacker et al., 2005). Interestingly, CORT is also able to bind ghrelin receptor (GHS-R1a) by displacing its natural ligand from its binding sites, capacity that is not shared by SST. In this sense, it has been recently demonstrated that interaction of CORT with ghrelin system precludes CORT to be a mere SST natural analog in the regulation of endocrine secretions. Indeed, our laboratory have recently demonstrated that CORT exhibits a stimulatory role on prolactin (PRL) secretion in primates and mice, which could be blocked *in vitro* by a GHS-R1a antagonist, a biological action that is not elicited by SST (Cordoba-Chacon et al., 2011).

## GHRELIN PLEIOTROPIC SYSTEM

The ghrelin gene, *GHRL*, encloses a 20-bp non-translated exon (Ex0), four coding exons (Ex1–4) and three introns, being a 28-aa native ghrelin peptide the predominant product of the 117-aa precursor pre-proghrelin (Kojima et al., 1999). Pre-proghrelin includes the signal peptide encoded by Ex1, and the coding sequence encoded by Ex2 and Ex3 which are the coding sequence of ghrelin (Seim et al., 2007). This transcript processing also generates different peptides or variants such as obestatin (of 23 aas), des-Glu14-ghrelin [matching to native ghrelin except for the deletion of one aa (Glu in the position 14)], etc. (Kineman et al., 2007; Seim et al., 2010).

Native ghrelin was originally isolated from the stomach of humans and rats based on its potent GH releasing activity (Kojima et al., 1999). Interestingly, native ghrelin has been the first natural hormone to be identified in which its third residue, usually a serine in mammals, contains the addition of a middle-chain fatty acid (*n*-octanoic acid) crucial for its biological activity. This post-translational modification is catalyzed by the ghrelin *O*-acyltransferase (GOAT; Gutierrez et al., 2008; Yang et al., 2008), a membrane bound *O*-acyltransferase located at the endoplasmic reticulum that uses fatty acids from the diet to fulfill its action (Kojima and Kangawa, 2005). Afterward, either acylated- or unacylated-proghrelin can be further processed by the prohormone convertase 1/3 (PC1/3) thus generating the acylated-ghrelin or its unacylated-ghrelin counterpart, a form of ghrelin initially considered as inactive (Zhu et al., 2006). Surprisingly, circulating unacylated-ghrelin levels are significantly higher than those of acylated-ghrelin in a proportion that depends on the study considered (Hosoda et al., 2000; Yoshimoto et al., 2002; Broglio et al., 2004; Liu et al., 2008).

Acylated-ghrelin elicits its biological actions through the GH-secretagogue receptor type-1a, GHS-R1a, previously known as an orphan receptor that mediates the GH-releasing effect of synthetic GH secretagogues, a group of peptide and non-peptide compounds with GH releasing properties. Currently, GHS-R1a is also called the ghrelin receptor based on the description of ghrelin as its natural ligand (Kojima et al., 1999).

The GHS-R gene consist of two exons whose transcription and processing originate two distinct forms of cDNAs: GHS-R1a, encoded by both exons, and a shorter form, GHS-R1b, derived from the exclusive transcription of the first exon (Howard et al., 1996; Kojima and Kangawa, 2005). The full-length of human GHS-R1a is a highly conserved protein of 366 aas that belong to the GPCR family containing seven putative membrane spanning alpha-helical segments and three intracellular and extracellular loops (Howard et al., 1996). GHS-R1a specifically recognizes the binding of acylated-ghrelin but not that of unacylated-ghrelin, whose specific receptor remains to be identified. In contrast, GHS-R1b isoform is an alternatively truncated variant of 289 aas that only possesses the first five transmembrane domains of GHS-1a (Howard et al., 1996; Kojima and Kangawa, 2005), and it was considered, until recently, to be a non-functional GHS-R isoform based on its inability to bind acylated-ghrelin. Interestingly, it has been recently described the interaction of GHS-R1b with GHS-R1a and other receptors to form heterodimers (Muccioli et al., 2007), as well as the heterodimerization of GHS-R1a with SST and dopamine receptors (Seim et al., 2010).

In terms of signal transduction, it has been described that GHS-R1a activation involves the participation of several signaling cascades including phospholipase C (PLC), protein kinase C (PKC), protein kinase A (Kojima and Kangawa, 2005) intracellular and extracellular Ca^2^^+^, and mitogen-activated protein kinases (Mousseaux et al., 2006; Camina et al., 2007).

Different components of the ghrelin system have been found to be ubiquitously represented in the organism. Specifically, ghrelin was originally described to be predominantly produced by endocrine cells of the stomach submucosa (Kojima et al., 1999). In addition, it was also documented to be produced at other portions of the gastrointestinal tract from the stomach to the colon and in a wide variety of peripheral tissues like the pancreas (Kojima et al., 1999; Date et al., 2000). It has also been showed the expression of ghrelin at different locations of the central nervous system (Ueberberg et al., 2009). The wider ghrelin tissue distribution is mimicked by that of GOAT, particularly in major ghrelin-secreting tissues (Gutierrez et al., 2008; Yang et al., 2008; Sakata et al., 2009) although GOAT transcripts appear to be much lower expressed than ghrelin transcripts. However, it has been documented that a small proportion of ghrelin expressing cells devoid of GOAT expression thus suggesting and supporting that unacylated-ghrelin might show independent biological actions to that described for ghrelin (Jeffery et al., 2011) and most probably by distinct receptor and mechanisms to those recruited by ghrelin (Toshinai et al., 2006; Sato et al., 2012).

On the other hand, GHS-R1a expression has been also widely detected in tissues or organs including pancreas, liver, stomach, adipose tissue, small and large intestine, immune system, and others (Gnanapavan et al., 2002; Sun et al., 2007; Ueberberg et al., 2009). In strikingly contrast, expression of GHS-R1b has been described to be even more extensive than that of GHS-R1a (Gnanapavan et al., 2002).

According to the wide tissue distribution of the different ghrelin system components, many physiological actions have been documented for this system. Based on the high conservation degree among species of ghrelin and its receptor sequences, it has been suggested that ghrelin system plays essential biological actions. In fact, and as mentioned earlier, acylated-ghrelin was initially identified based on its ability to stimulate GH release upon GHS-R1a coupling in a similar manner to that described for GH secretagogues (Kojima et al., 1999). Also involving GHS-R1a activation, ghrelin elicits an orexigenic role by promoting food intake or appetite (Wren et al., 2000; Druce et al., 2005, 2006) and weight gain and adiposity (Tschop et al., 2000; Wren et al., 2000, 2001). Ghrelin has also shown regulatory properties on glucose and energy homeostasis that will be revised separately. In addition to its metabolic actions, ghrelin has also been reported to exert potent anti-inflammatory actions in therapeutically relevant models of arthritis or inflammatory bowel disease (Gonzalez-Rey et al., 2006; Gonzalez-Rey and Delgado, 2008) and also influence atherogenesis (Kadoglou et al., 2012). Furthermore, ghrelin shows favorable effects on cardiovascular and gastroenteropancreatic physiology (Isgaard and Granata, 2011), as well as on the modulation of the immune system (Souza-Moreira et al., 2011). At the central nervous system, ghrelin influences memory, learning, and behavior functions (Asakawa et al., 2001; Broglio et al., 2003). Besides the above-described stimulatory effect on GH release, pituitary function is also regulates by ghrelin by stimulating the release of PRL and adrenocorticotropin (van der Lely et al., 2004; Coiro et al., 2005).

On the other hand, unacylated-ghrelin, plays both equal or opposite biological actions to that above described for acyl-ghrelin, most probably through its coupling to a still unknown receptor different from GHS-R1a, as it has been recently suggested and documented (Toshinai et al., 2006; Lear et al., 2010; Togliatto et al., 2010). Moreover, regulation of unacylated-ghrelin secretion under food restriction condition has been reported, thus supporting a not passive role for this unmodified peptide (Kirchner et al., 2009).

Regarding obestatin, this 23-aa peptide is mainly produced in the stomach and at lower level in the pancreas, spleen, testis, duodenum, jejunum, colon, and mammary gland (Ren et al., 2009). To date, although obestatin receptor remains unknown, it has been proposed that GPR39 or glucagon-like peptide-1 receptor might be potential receptors for obestatin (Granata et al., 2008; Ren et al., 2009).

## ACTIONS OF SST/CORT SYSTEM AT THE ENDOCRINE PANCREAS

Somatostatin has long been known to potently modulate pancreatic function by playing a regulatory role on insulin and glucagon secretion. This tight regulation is accomplished by the coordinated action of SST biologically active forms. In this sense, SST-14 is the major product released by adult pancreatic delta-cells, whose contribution to total circulating SST accounts for nearly 5%. SST-14 immediate actions imply the paracrine regulation of other pancreatic endocrine cells. In addition, endocrine pancreatic function is also under the control of the major circulating SST form, SST-28. In this context, it has been documented that SST-14 and SST-28 differently target pancreatic alpha- or beta-cells. Accordingly, SST-14 has been associated with the inhibition of glucagon secretion while SST-28 seems preferentially to inhibit insulin secretion (Strowski and Blake, 2008). In either case, inhibitory action of SST on both insulin and glucagon release would contribute to preserve glucose homeostasis which, in turn, retrospectively regulates SST plasma concentration. For this reason, during fasting, plasma SST level is low and increases up to twofold after meals. In such hyperglycemic conditions, insulin induces SST release and consequently shuts down its own release as a protective mechanism to prevent a rapid hypoglycemia at the post-prandial state. In contrast, SST release is also increased at low plasma glucose concentration as a consequence of the coordinated action of glucagon and L-glutamate, a co-secretion product of alpha-cells (Strowski and Blake, 2008).

The SST action on pancreatic hormones is mediated by its interaction with the different receptor isoforms, however, conflicting data about receptor expression at the pancreatic level have been published which, might be related with the different method used and/or species analyzed. In general, it is likely that endocrine pancreas expresses all five sst isoforms, being probably sst1, sst2, and sst5 those expressed in a predominant manner. Specifically, it has been demonstrated by RT-PCR that rodent pancreas expresses all sst isoforms except sst4. In turn, by double immunohistochemistry methods, it has been confirmed the expression of sst2 and sst5 in both rodent beta- and delta-cells. In humans, it has been reported a predominant expression of sst2 in alpha-cells, sst1 and sst5 in beta-cells, and sst5 in delta-cells while there is not consistent data on the expression of the rest of ssts (Strowski and Blake, 2008; Gahete et al., 2010).

In addition, and in order to ascertain the subtype receptors underlying the inhibitory effect of SST on pancreatic hormones, specific receptor agonists, and antagonists as well as *knock out* mouse models with deletion of the different ssts isoforms have been employed. These issues have been amply revised elsewhere (Strowski and Blake, 2008) and briefly, it appears that sst2 seems to mediate glucagon inhibition and sst5 looks as the main receptor mediating insulin inhibition in rodents. More recently, high expression of sst3 has been also demonstrated in mouse pancreatic islets (Regard et al., 2008). In human, by contrast, sst2 looks to be the main receptor mediating both insulin and glucagon release, although sst1 and sst5 also participate in the regulation of insulin secretion (Gahete et al., 2010). In summary, several sst isoforms would mediate the inhibitory action of SST on insulin and glucagon secretion through a mechanism that indubitably is species-dependent, and will essentially depend on the receptor expression pattern.

In relation to CORT and its role on pancreatic function, only a few studies are available. Particularly and similarly to SST, CORT expression has been reported at the endocrine pancreas and essentially mimics its inhibitory action on insulin secretion under physiological and certain pathological conditions (Grottoli et al., 2006; Broglio et al., 2008), although the molecular mechanism underlying such inhibitory action is still unclear. In addition, it should be highlighted that CORT is also able to elicit distinct functions to that showed by SST mainly through its coupling to the ghrelin receptor (GHS-R1), as it has been recently documented for others endocrine secretions (Cordoba-Chacon et al., 2011).

## ACTIONS OF GHRELIN SYSTEM AT THE ENDOCRINE PANCREAS

After ghrelin discovery, it was reported that pancreatic epsilon-cells are the major source of ghrelin forms during fetal life until early post-natal period (Wierup et al., 2002; Chanoine and Wong, 2004). After this period, the major source of ghrelin production is the stomach submucosa (Kojima et al., 1999) while, the pancreas turns on a secondary source of ghrelin production with low level of ghrelin receptor expression (Veldhuis and Bowers, 2010). At the pancreas, the major source of ghrelin resides into epsilon-cells (Wierup et al., 2002, 2004; Prado et al., 2004) although it appears that is also produced by beta-cells in humans (Volante et al., 2002) and by glucagon-producing alpha-cells in human and rats (Date et al., 2002). In any case and in terms of ghrelin production, it has been described that during adult life the 65–90% of circulating ghrelin corresponds to that synthesized and released by the stomach, being the rest derived from other tissues including the pancreas and the intestine (Al Massadi et al., 2011).

Endocrine ghrelin actions at the pancreas involve inhibition of SST release by delta-cells, and stimulation of glucagon release by alpha-cells (Qader et al., 2005, 2008; Veldhuis and Bowers, 2010; Chuang et al., 2011) as well as inhibition of pancreatic polypeptide release by PP cells (Qader et al., 2008; Kumar et al., 2010), being all cells types in which GHS-R expression has been documented (Wierup et al., 2002, 2004; Kageyama et al., 2005; Dezaki et al., 2008; Granata et al., 2010a). On the other hand, SST and glucagon have also been shown to elicit a reciprocal modulation of ghrelin production (**Figure 2**). Furthermore, it has been reported that insulin and SST inhibit ghrelin release while glucagon inhibits its secretion in rodent although stimulate ghrelin release in humans (Qader et al., 2008).

Although ghrelin effect on insulin secretion is supported by an increasing number of reports, its precise role is nevertheless controversial since either stimulatory or inhibitory actions has been reported depending on the ghrelin doses used and/or experimental conditions employed as recently reviewed by Granata et al. (2010a), 2010b. Specifically, the ghrelin stimulatory effect of insulin release is mainly mediated by an increase of cytosolic Ca^2^^+^ upon GHS-R activation, while the participation of a different receptor has been proposed based on the observed stimulatory action of both acylated- and unacylated-ghrelin on insulin release in a hamster beta-cell line devoid of GHS-R expression (Granata et al., 2007). In addition, it has also been described that ghrelin administration increases insulin release in rats under conditions of low blood insulin as a consequence of a 90% pancreatectomy (Kerem et al., 2009). Reciprocally, insulin inhibits ghrelin expression (Veldhuis and Bowers, 2010) and more recently it has been proposed that insulin might act as an inhibitor of pancreatic ghrelin activation by inhibition of GOAT expression (An et al., 2011).

In clear contrast to the above stimulatory role of ghrelin on insulin secretion, the ghrelin inhibitory action has been prevalently reported and examined in several biological and animal models including cultured pancreata, cultured islets and heterologous cell models (Granata et al., 2010a; Dezaki et al., 2011), as well as by using several methodological approaches. Overall, it has been described that ghrelin administration significantly reduces insulin secretion (Broglio et al., 2001; Tong et al., 2010), and this action was blocked in the presence of a higher dose of unacylated-ghrelin, suggesting the participation of a receptor distinct to GHS-R on insulin secretion modulation (Qader et al., 2008). Reduction of insulin level by ghrelin administration triggers a concomitant elevation of blood glucose levels in a dose-dependent manner as well as deterioration of insulin sensitivity during glucose tolerance, as it have been described in both humans and rodents (Korbonits and Grossman, 2004; Dezaki et al., 2008; Tong et al., 2010; Sato et al., 2012). The hyperglycemic action of ghrelin, but not by unacylated-ghrelin in rodents, was blocked by simultaneous administration of GHS-R antagonist thus revealing the specific participation of GHS-R in the hyperglycemic role of ghrelin (Dezaki et al., 2008). In addition, GHS-R deletion also reduces blood glucose level and significantly enhances insulin sensitivity (Longo et al., 2008; Qi et al., 2011). Importantly, the involvement of GH (a long time known hyperglycemic hormone) has been excluded from the hyperglycemic effect of ghrelin administration based on elevated plasma glucose levels observed in response to exogenous ghrelin administration in GH-deficient mice (Dezaki et al., 2008) and subjects with GH-deficiency (Vestergaard et al., 2008). Similarly, ghrelin hyperglycemic effect have been reported to be independent of an insulin resistance induction as evidenced by insulin and glucose tolerance tests after ghrelin administration (Dezaki et al., 2008).

In order to ascertain whether the insulinostatic action of ghrelin is due to the peptide derived from the stomach or other sources as the pancreas, GHS-R antagonist was administered to gastrectomized animals and a significant increase in insulin secretion was observed in a similar extend to that observed in normal rats. This observation suggests that intra-islet ghrelin may locally act on insulin production regulation (Dezaki et al., 2007). However, Bando et al. (2012) recently reported that intra-islet ghrelin does not play a major local role on the regulation of insulin release *in vivo*, based on their findings on transgenic mice in which ghrelin and GOAT were overexpressed in beta-cells. The discrepancy between these two later studies may reside on the different ghrelin concentration reached at the surrounding microenvironment of beta-cells.

In addition to the regulatory role of ghrelin on pancreatic function, it has also been described that acylated- and unacylated-ghrelin as well as obestatin elicit a protective role by preventing apoptosis on pancreatic islet in rodents, humans, and several beta-cell lines (Granata et al., 2012b). In this sense, it has been reported that beta-cell destruction elicited by streptozotocin administration was precluded by ghrelin by increasing both beta-cell mass and insulin release in rats (Irako et al., 2006). Furthermore, ghrelin and obestatin also protect against apoptosis induced by serum starvation and cytokines in both human islets and beta-cell lines (Granata et al., 2007, 2008, 2010a). In well agreement with this, ghrelin and obestatin exert their mitogenic effect by increasing the number of beta-cells in 90% pancreatectomized rats (Kerem et al., 2009) and in a hamster beta-cells line (Granata et al., 2010a), proliferative action that was blocked by administration of ghrelin antagonist or ghrelin antibody (Kerem et al., 2009; Granata et al., 2010a). These findings suggest that a cross-talk between ghrelin and obestatin may exist (Granata et al., 2008).

## ROLE OF SST/CORT SYSTEM IN T2DM AND OBESITY

**FIGURE 1:**
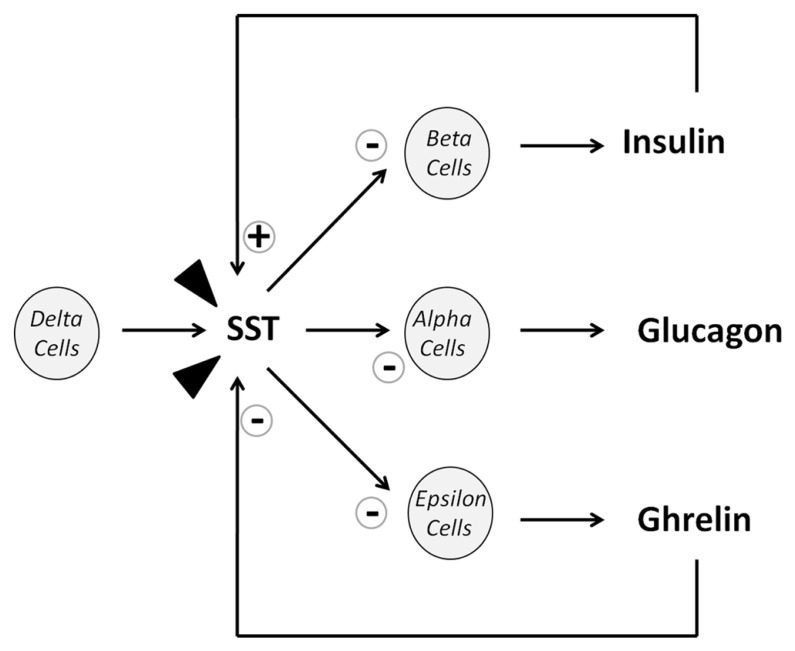
**Diagram of SST actions on islet cell release.** Arrow heads represent SST from extrapancreatic origins (mostly intestinal).

%They are not many reports on the role of SST/CORT in situations with altered metabolic conditions. In this sense, an increase in the number of SST-producing cells in T2DM patients has been recently described, although circulating SST remains in the same level to that depicted by control subjects. However, in an experimental model of obese and spontaneously diabetic mice, SST content is significantly increased (Strowski and Blake, 2008). In this scenario, the well established inhibitory actions of SST on pancreatic function, particularly on insulin and glucagon secretion (**Figure 1**) as well as its inhibitory action on intestinal glucose absorption, predicted its use as a key tool to potentially regulate glucose homeostasis and insulin sensitivity in diabetes and obesity (Hansen et al., 2004; Tzotzas et al., 2008). Indeed, initial studies evaluated SST role on insulin hypersecretion as well as in hyperinsulinemia associated with obesity, two conditions that were described to induce insulin resistance (Janson and Oberg, 1999; Boehm, 2003). Consequently, significant reductions in body weight and insulin release as well as an improvement of insulin sensitivity were observed in obese patients treated with synthetic SST analogs (Boehm and Lustig, 2002; Velasquez-Mieyer et al., 2004; Lustig et al., 2006; Tzotzas et al., 2008), which were originally developed as a consequence of the SST short half-life. Similarly to SST, it has also been reported an inhibitory action of CORT on insulin release in patients with acromegaly or prolactinoma (Grottoli et al., 2005). In relation to CORT, and based on its described anti-inflammatory properties, it would be of interest to explore its role on the inflammatory signaling that occurs during obesity conditions.

**FIGURE 2:**
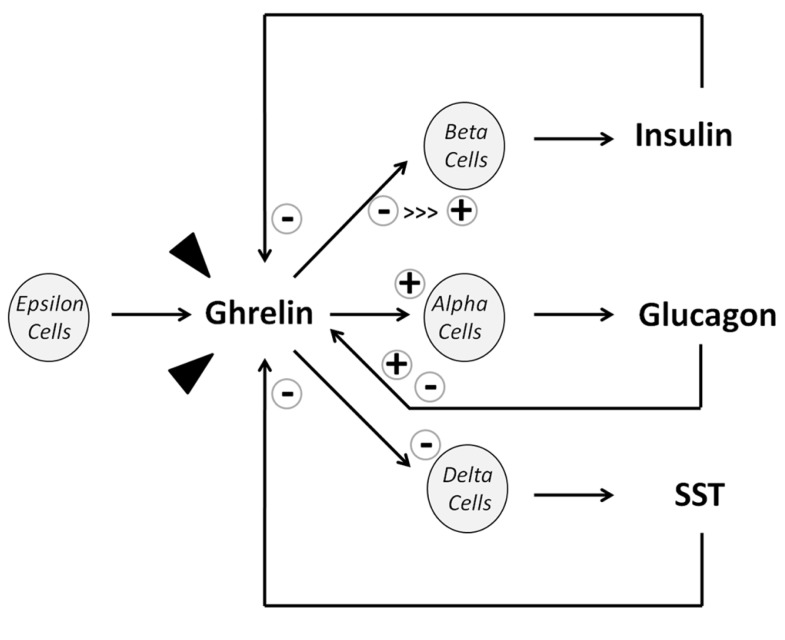
** Diagram of ghrelin actions on islet cell release.** Arrow heads represent ghrelin from extrapancreatic origins (mostly stomach). “>>>” denotes that the majority of published studies describe an inhibitory effect of ghrelin on insulin release.

More recently, the effect of a multi-ligand SST analog (pasireotide) on hormones that mediate glucose homeostasis has been described in healthy volunteers (Golor et al., 2012; Shenouda et al., 2012). Accordingly, based on the high binding affinity for four of the five SST receptor subtypes (sst1–3, and sst5) elicited by pasireotide, it has been administered to healthy subjects and an elevation of blood glucose has been observed mainly as a consequence of its inhibitory action on both insulin and glucagon release (Shenouda et al., 2012). Similar hyperglycemic effect of pasireotide has been observed in clinical trials in which pasireotide administration was evaluated on patients with endocrine pathologies as Cushing’s disease, acromegaly, and neuroendocrine tumors (NETs). In these pathologies, hyperglycemia might be further worsened in base to their inherent hormonal nature (Boscaro et al., 2009; Petersenn et al., 2010; Colao et al., 2012). Furthermore, variable sst1–5 expression has been extensively documented and consequently SST analogs have been classically used after adenoma or tumor resection. Lastly, SST analogs have been used as a first option therapy in selected patients upon its ability to reduce hormonal release and/or to inhibit tumor mass progression. In these cases, alterations in glucose homeostasis as well as impaired insulin resistance has been well documented as a secondary effect to their characteristic underlying hormonal secretion (Resmini et al., 2009; Shenouda et al., 2012).

In different series of acromegalic patients, a significant prevalence of impaired glucose metabolism and diabetes mellitus has been described, a consequence that is believed to be originated by the excess GH (van der Hoek et al., 2005; Espinosa-de-los-Monteros et al., 2011; Fieffe et al., 2011; Kinoshita et al., 2011). In such GH hypersecreting conditions, the universal sst binding profile of pasireotide and its higher efficacy by lowering GH release more significantly than sst2 preferential or specific SST analogs (octreotide and lanreotide) granted its therapeutical use. However, as mentioned above in these subjects, the risk of hyperglycemia increases as a common adverse event originated by the normalization of GH and IGF-1 levels as well as the inhibition of insulin release by SST administration (Resmini et al., 2009).

Somatostatin analogs have also been applied in the management of NET suffering patients in which, similarly to acromegalic individuals, an altered glucose tolerance has been documented (Cirillo, 2010). Such altered glucose tolerance may occur as a consequence of hormonal dysregulation or pancreatic resection (Resmini et al., 2009; Jawiarczyk et al., 2012).

Hypercortisolism condition, a distinctive characteristic of Cushing’s disease, also leads to hyperglycemia and reduced glucose tolerance and as a result, in an increase of the prevalence of diabetes in this pathology (Mazziotti et al., 2009, 2011; Resmini et al., 2009). Furthermore, hyperglycemia persists even when cortisol level declines by administration of SST analogs, i.e., pasireotide as it has been recently reported (Colao et al., 2012).

In sum, hyperglycemia conditions occur in an elevated proportion of individuals suffering of acromegaly, Cushing’s disease or NET and accordingly, it has been proposed that regular blood glucose testing and insulin analogs will be required, particularly when SST analogs are therapeutically used in these pathologies (Resmini et al., 2009; Colao et al., 2012).

## ROLE OF GHRELIN SYSTEM IN T2DM AND OBESITY

As mentioned earlier, a growing body of studies supports the inhibitory role of ghrelin on insulin release *in vivo* and *in vitro* and its influence on glucose tolerance. Accordingly, it has been proposed that the antagonism of ghrelin system components could improve glucose homeostasis and/or beta-cell function under certain metabolic disorders as T2DM, a complex disease with a strong genetic, behavioral, and environmental background that is characterized by two distinctively conditions: insulin resistance and progressive beta-cell dysfunction. In T2DM, beta-cells become unable to adequately increase insulin release to compensate insulin resistance and consequently leading to a situation of hyperglycemia. It is well known the close association between T2DM and obesity in terms of metabolic imbalance and their common features, insulin resistance, in which ghrelin system could be of relevance based on its ability to modulate both glucose homeostasis and weight loss (Esler et al., 2007).

Under normal metabolic conditions, circulating ghrelin and plasma glucose concentrations are inversely related. In fact, ghrelin levels are increased under fasting conditions or immediately before meals and significantly decreased after feeding (Angelidis et al., 2010). Obviously, the meal-related pattern of ghrelin is also opposite to that depicted by insulin and consequently the fall of ghrelin at post-prandial state has been argued to partially depend on the rise of insulin release after food intake (Solomon et al., 2008). Accordingly, the tight relationship between ghrelin and insulin also relies in the general assumption that insulin elicits a negative action on both plasma acylated- and unacylated-ghrelin concentration (Saad et al., 2002; McLaughlin et al., 2004), while administration of acylated-ghrelin results in insulin resistance (Gauna and van der Lely, 2005).

In subjects affected by T2DM and consequently resistant to insulin, it has been showed that blood ghrelin concentration was chronically lower than that observed in healthy subjects even when age, sex, and body mass index (BMI) were adjusted, probably as a direct effect of insulin on ghrelin-producing cells (Poykko et al., 2003; Angelidis et al., 2010; Verhulst and Depoortere, 2012). Based on the influence of ghrelin on insulin release and glucose homeostasis, it has been suggested that ghrelin antagonism could be of interest to treat T2DM and related metabolic pathologies. In this context, it has been shown that deletion of ghrelin gene promotes insulin release and ameliorates glucose intolerance and hyperglycemia in a diabetic and obese mice model (Sun et al., 2006). In well agreement with this, GHS-R ablation also improves insulin sensitivity (Gomez et al., 2009). Likewise, it has also been documented an improvement of glucose homeostasis in streptozotocin-induced diabetic rats treated with obestatin (Granata et al., 2010b) as well as a reduction in insulin resistance in mice fed with a high fat diet (Granata et al., 2012a).

On the other hand, obesity conditions have been associated with some ghrelin and GHS-R gene variations although some discrepancies exist depending on studies and population considered (Pantel et al., 2006; Liu et al., 2007; Ukkola, 2011). In this sense, available data are still inconclusive and might be limited by some relatively small analyzed cohorts that might restrict the power of association.

However, it has been well documented that under obesity conditions plasma ghrelin levels negatively correlate with BMI and consequently with factors or parameters that are elevated in obesity such as insulin, leptin, and fat mass (Tschop et al., 2001). In this sense a chronic lower ghrelin plasma concentration in obese children and adults has been reported in comparison with those of age-matched lean controls (Tschop et al., 2001; Reinehr et al., 2008; Schellekens et al., 2010). Similar data have been cited for Pima Indians, a population reported with the highest prevalence rates of obesity and T2DM when compared with Caucasians (Tschop et al., 2001). Similarly to that reported under normal metabolic condition, ghrelin also elicits a meal-related pattern although under obesity conditions the fall of ghrelin level at post-prandial state is less pronounced. Such downregulation might be a consequence of elevated fasting insulin or leptin levels observed in obesity (Baragli et al., 2011). In this sense, it has also been suggested that the decreased secretion of ghrelin, could be responsible for the concomitant decreased levels of circulating GH observed in obese individuals (Maccario et al., 2000; Tschop et al., 2001). More recently, the decreased ghrelin concentration observed in obesity could be an adaptive mechanism to maintain energy homeostasis has also been proposed (Tschop et al., 2001). In rodents, diminished ghrelin levels have been found at tissue level as well as a significant reduction in plasma ghrelin concentration and synthesis in obesity conditions induced by diet (Sahin et al., 2011; Aydin et al., 2012).

Targeting ghrelin system components by different pharmacological, immunological, and genetic approaches have been addressed in order to promote weight loss and to improve obesity conditions (i.e., insulin resistance). Thus, some studies evaluated pharmacological approaches to block or neutralize either ghrelin or its receptor under diet-induced obesity and how these methods ameliorate obesity condition by reducing appetite or food intake, and ultimately inducing weight loss (Wortley et al., 2005; Schellekens et al., 2010; Briggs and Andrews, 2011). Another set of studies, examined the protection of ghrelin system against rapid weight gain by exposure to a high fat diet by knocking out either ghrelin (Wortley et al., 2005) or GHS-R (Castaneda et al., 2010). In these studies, an improvement of glucose tolerance was observed even though there is no effect on body weight (Zigman et al., 2005; Longo et al., 2008). Similar data were obtained in a genetically obese mice model (ob/ob; leptin deficient) in which the improvement of insulin sensitivity and glucose homeostasis was attributed to ghrelin although the obese phenotype remains unchanged (Sun et al., 2006).

In addition, it has also been proposed that GOAT by a specific inhibitor could be a potential treatment against obesity by inhibiting ghrelin acylation and consequently avoid weight gain (Gualillo et al., 2008; Yang et al., 2008; Gomez et al., 2009; Barnett et al., 2010).

On the other hand, diet-induced weight loss elicits an increase in circulating ghrelin levels thus normalizing them until near optimal concentration, rise that probably may hamper the sustained weight loss (Cummings et al., 2002; Hansen et al., 2002). In cases of morbid obesity, a more drastic method as bariatric surgery has been employed in order to reduce metabolic complications associated to obesity. Interestingly and contrary to that reported in diet-induced weight loss, after bariatric surgery ghrelin level significantly decreases and insulin sensitivity is rapidly restored, thus improving the associated diabetic state (Beckman et al., 2010; Hillman et al., 2011). In this context, sustained low ghrelin level reached by this surgical procedure precludes or delays weight gain by reducing hunger, an effect that is not observed in procedures as weight loss by diet modification in which ghrelin levels gradually normalizes with a consequent weight gain. On the other hand, in morbid obese patients, it has been suggested that equimolar administration of acylated- and unacylated-ghrelin also improve insulin sensitivity (Kiewiet et al., 2009).

Unfortunately, there is not yet a ghrelin system based therapy that ensures a sustained weight loss, although ghrelin antagonist and/or GOAT inhibitors may be considerate good therapeutic candidates for the treatment of T2DM and obesity.

## CONCLUSION

The complex relationship of ligand–receptor architecture of SST/CORT and ghrelin systems is complemented by the functional relevance of their common tissue targets. Indeed, besides their opposite influence on GH release at the pituitary level, SST/CORT and ghrelin systems act on the same cellular target to influence common and relevant biological actions as it is the case of their interactions on beta cell function and survival as well as on glucose homeostasis and insulin resistance. However, the underlying molecular mechanisms of these actions have not been fully elucidated yet. Interestingly, different and severe metabolic dysfunctions as T2DM and obesity have been described to modulate their circulating levels and the expression of some components of both systems at hypothalamic, pituitary, or pancreatic level. In sum, there are an increasing number of evidences that support a potential contribution of SST/CORT and ghrelin system components in the endocrine pancreas dysfunction in prevalent neuroendocrine-metabolic pathologies (T2DM and obesity) which suggest that these systems could be considered as future valuable therapeutic targets for the prevention or treatment of such metabolic disorders. In this sense, future research will be of particular importance in order to ascertain whether SST/CORT and ghrelin systems and its receptors will act at pancreatic islets under physiological and pathological conditions. Likewise, the underlying molecular mechanisms as well as the precise role and the contribution of islet derived-SST, -CORT, and -ghrelin in the pancreatic endocrine deregulation and/or the resulting insulin resistance under severe metabolic conditions should be investigated.

## Conflict of Interest Statement:

The authors declare that the research was conducted in the absence of any commercial or financial relationships that could be construed as a potential conflict of interest.

## References

[B1] Al Massadi O. Tschop M. H. Tong J. (2011). Ghrelin acylation and metabolic control. *Peptides* 32 2301–2308 21893140 10.1016/j.peptides.2011.08.020

[B2] An W. Li Y. Xu G. Zhao J. Xiang X. Ding L. Li J. Guan Y. Wang X. Tang C. Li X. Mulholland M. Zhang W. (2011). Modulation of ghrelin *O*-acyltransferase expression in pancreatic islets. *Cell. Physiol. Biochem.* 26 707–716 21063108 10.1159/000322338 PMC3048940

[B3] Angelidis G. Valotassiou V. Georgoulias P. (2010). Current and potential roles of ghrelin in clinical practice. *J. Endocrinol. Invest.* 33 823–838 21293171 10.1007/BF03350350

[B4] Asakawa A. Inui A. Kaga T. Yuzuriha H. Nagata T. Fujimiya M. Katsuura G. Makino S. Fujino M. A. Kasuga M. (2001). A role of ghrelin in neuroendocrine and behavioral responses to stress in mice. *Neuroendocrinology* 74 143–147 11528215 10.1159/000054680

[B5] Aydin S. Sahin I. Ozkan Y. Dag E. Gunay A. Guzel S. P. Catak Z. Ozercan M. R. (2012). Examination of the tissue ghrelin expression of rats with diet-induced obesity using radioimmunoassay and immunohistochemical methods. *Mol. Cell. Biochem.* 365 165–173 22350756 10.1007/s11010-012-1256-4

[B6] Ballian N. Brunicardi F. C. Wang X. P. (2006). Somatostatin and its receptors in the development of the endocrine pancreas. *Pancreas* 33 1–12 16804406 10.1097/01.mpa.0000226894.16817.e8

[B7] Bando M. Iwakura H. Ariyasu H. Hosoda H. Yamada G. Hosoda K. Adachi S. Nakao K. Kangawa K. Akamizu T. (2012). Transgenic overexpression of intraislet ghrelin does not affect insulin secretion or glucose metabolism in vivo. *Am. J. Physiol. Endocrinol. Metab.* 302 E403–E408 22114024 10.1152/ajpendo.00341.2011

[B8] Baragli A. Lanfranco F. Allasia S. Granata R. Ghigo E. (2011). Neuroendocrine and metabolic activities of ghrelin gene products. *Peptides* 32 2323–2332 22056513 10.1016/j.peptides.2011.10.024

[B9] Barnett B. P. Hwang Y. Taylor M. S. Kirchner H. Pfluger P. T. Bernard V. Lin Y. Y. Bowers E. M. Mukherjee C. Song W. J. Longo P. A. Leahy D. J. Hussain M. A. Tschop M. H. Boeke J. D. Cole P. A. (2010). Glucose and weight control in mice with a designed ghrelin *O*-acyltransferase inhibitor. *Science* 330 1689–1692 21097901 10.1126/science.1196154 PMC3068526

[B10] Beckman L. M. Beckman T. R. Earthman C. P. (2010). Changes in gastrointestinal hormones and leptin after Roux-en-Y gastric bypass procedure: a review. *J. Am. Diet. Assoc.* 110 571–584 20338283 10.1016/j.jada.2009.12.023 PMC4284064

[B11] Boehm B. O. (2003). The therapeutic potential of somatostatin receptor ligands in the treatment of obesity and diabetes. *Expert Opin. Investig. Drugs* 12 1501–1509 10.1517/13543784.12.9.1501 12943494

[B12] Boehm B. O. Lustig R. H. (2002). Use of somatostatin receptor ligands in obesity and diabetic complications. *Best Pract. Res. Clin. Gastroenterol.* 16 493–509 12079271 10.1053/bega.2002.0320

[B13] Boscaro M. Ludlam W. H. Atkinson B. Glusman J. E. Petersenn S. Reincke M. Snyder P. Tabarin A. Biller B. M. Findling J. Melmed S. Darby C. H. Hu K. Wang Y. Freda P. U. Grossman A. B. Frohman L. A. Bertherat J. (2009). Treatment of pituitary-dependent Cushing's disease with the multireceptor ligand somatostatin analog pasireotide (SOM230): a multicenter, phase II trial. *J. Clin. Endocrinol. Metab.* 94 115–122 18957506 10.1210/jc.2008-1008

[B14] Brazeau P. Vale W. Burgus R. Ling N. Butcher M. Rivier J. Guillemin R. (1973). Hypothalamic polypeptide that inhibits the secretion of immunoreactive pituitary growth hormone. *Science* 179 77–79 4682131 10.1126/science.179.4068.77

[B15] Briggs D. I. Andrews Z. B. (2011). Metabolic status regulates ghrelin function on energy homeostasis. *Neuroendocrinology* 93 48–57 21124019 10.1159/000322589

[B16] Broglio F. Arvat E. Benso A. Gottero C. Muccioli G. Papotti M. Van Der Lely A. J. Deghenghi R. Ghigo E. (2001). Ghrelin, a natural GH secretagogue produced by the stomach, induces hyperglycemia and reduces insulin secretion in humans. *J. Clin. Endocrinol. Metab.* 86 5083–5086 11600590 10.1210/jcem.86.10.8098

[B17] Broglio F. Gottero C. Arvat E. Ghigo E. (2003). Endocrine and non-endocrine actions of ghrelin. *Horm. Res.* 59 109–117 12637790 10.1159/000069065

[B18] Broglio F. Gottero C. Prodam F. Gauna C. Muccioli G. Papotti M. Abribat T. Van Der Lely A. J. Ghigo E. (2004). Non-acylated ghrelin counteracts the metabolic but not the neuroendocrine response to acylated ghrelin in humans. *J. Clin. Endocrinol. Metab.* 89 3062–3065 15181099 10.1210/jc.2003-031964

[B19] Broglio F. Grottoli S. Arvat E. Ghigo E. (2008). Endocrine actions of cortistatin: in vivo studies. *Mol. Cell. Endocrinol.* 286 123–127 18281148 10.1016/j.mce.2007.12.012

[B20] Camina J. P. Lodeiro M. Ischenko O. Martini A. C. Casanueva F. F. (2007). Stimulation by ghrelin of p42/p44 mitogen-activated protein kinase through the GHS-R1a receptor: role of G-proteins and beta-arrestins. *J. Cell. Physiol.* 213 187–200 17525997 10.1002/jcp.21109

[B21] Castaneda T. R. Tong J. Datta R. Culler M. Tschop M. H. (2010). Ghrelin in the regulation of body weight and metabolism. *Front. Neuroendocrinol.* 31 44–60 19896496 10.1016/j.yfrne.2009.10.008

[B22] Chanoine J. P. Wong A. C. (2004). Ghrelin gene expression is markedly higher in fetal pancreas compared with fetal stomach: effect of maternal fasting. *Endocrinology* 145 3813–3820 15142981 10.1210/en.2004-0053

[B23] Chuang J. C. Sakata I. Kohno D. Perello M. Osborne-Lawrence S. Repa J. J. Zigman J. M. (2011). Ghrelin directly stimulates glucagon secretion from pancreatic alpha-cells. *Mol. Endocrinol.* 25 1600–1611 21719535 10.1210/me.2011-1001 PMC3165914

[B24] Cirillo F. (2010). Role of somatostatin analogs in the management of neuroendocrine tumors. *Tumori* 96 191–197 20572573 10.1177/030089161009600202

[B25] Coiro V. Saccani-Jotti G. Minelli R. Melani A. Milli B. Manfredi G. Volpi R. Chiodera P. (2005). Adrenocorticotropin/cortisol and arginine-vasopressin secretory patterns in response to ghrelin in normal men. *Neuroendocrinology* 81 103–106 15860925 10.1159/000085541

[B26] Colao A. Petersenn S. Newell-Price J. Findling J. W. Gu F. Maldonado M. Schoenherr U. Mills D. Salgado L. R. Biller B. M. (2012). A 12-month phase 3 study of pasireotide in Cushing's disease. *N. Engl. J. Med.* 366 914–924 22397653 10.1056/NEJMoa1105743

[B27] Cordoba-Chacon J. Gahete M. D. Pozo-Salas A. I. Martinez-Fuentes A. J. de Lecea L. Gracia-Navarro F. Kineman R. D. Castano J. P. Luque R. M. (2011). Cortistatin is not a somatostatin analogue but stimulates prolactin release and inhibits GH and ACTH in a gender-dependent fashion: potential role of ghrelin. *Endocrinology* 152 4800–4812 21971153 10.1210/en.2011-1542 PMC3230064

[B28] Cummings D. E. Weigle D. S. Frayo R. S. Breen P. A. Ma M. K. Dellinger E. P. Purnell J. Q. (2002). Plasma ghrelin levels after diet-induced weight loss or gastric bypass surgery. *N. Engl. J. Med.* 346 1623–1630 12023994 10.1056/NEJMoa012908

[B29] Date Y. Kojima M. Hosoda H. Sawaguchi A. Mondal M. S. Suganuma T. Matsukura S. Kangawa K. Nakazato M. (2000). Ghrelin, a novel growth hormone-releasing acylated peptide, is synthesized in a distinct endocrine cell type in the gastrointestinal tracts of rats and humans. *Endocrinology* 141 4255–4261 11089560 10.1210/endo.141.11.7757

[B30] Date Y. Nakazato M. Hashiguchi S. Dezaki K. Mondal M. S. Hosoda H. Kojima M. Kangawa K. Arima T. Matsuo H. Yada T. Matsukura S. (2002). Ghrelin is present in pancreatic alpha-cells of humans and rats and stimulates insulin secretion. *Diabetes* 51 124–129 11756331 10.2337/diabetes.51.1.124

[B31] de Lecea L. Criado J. R. Prospero-Garcia O. Gautvik K. M. Schweitzer P. Danielson P. E. Dunlop C. L. Siggins G. R. Henriksen S. J. Sutcliffe J. G. (1996). A cortical neuropeptide with neuronal depressant and sleep-modulating properties. *Nature* 381 242–245 8622767 10.1038/381242a0

[B32] Dezaki K. Damdindorj B. Sone H. Dyachok O. Tengholm A. Gylfe E. Kurashina T. Yoshida M. Kakei M. Yada T. (2011). Ghrelin attenuates cAMP–PKA signaling to evoke insulinostatic cascade in islet beta-cells. *Diabetes* 60 2315–2324 21788571 10.2337/db11-0368 PMC3161328

[B33] Dezaki K. Kakei M. Yada T. (2007). Ghrelin uses Galphai2 and activates voltage-dependent K+ channels to attenuate glucose-induced Ca2+ signaling and insulin release in islet beta-cells: novel signal transduction of ghrelin. *Diabetes* 56 2319–2327 17575083 10.2337/db07-0345

[B34] Dezaki K. Sone H. Yada T. (2008). Ghrelin is a physiological regulator of insulin release in pancreatic islets and glucose homeostasis. *Pharmacol. Ther.* 118 239–249 18433874 10.1016/j.pharmthera.2008.02.008

[B35] Druce M. R. Neary N. M. Small C. J. Milton J. Monteiro M. Patterson M. Ghatei M. A. Bloom S. R. (2006). Subcutaneous administration of ghrelin stimulates energy intake in healthy lean human volunteers. *Int. J. Obes. (Lond.)* 30 293–296 16247504 10.1038/sj.ijo.0803158

[B36] Druce M. R. Wren A. M. Park A. J. Milton J. E. Patterson M. Frost G. Ghatei M. A. Small C. Bloom S. R. (2005). Ghrelin increases food intake in obese as well as lean subjects. *Int. J. Obes. (Lond.)* 29 1130–1136 15917842 10.1038/sj.ijo.0803001

[B37] Esler W. P. Rudolph J. Claus T. H. Tang W. Barucci N. Brown S. E. Bullock W. Daly M. Decarr L. Li Y. Milardo L. Molstad D. Zhu J. Gardell S. J. Livingston J. N. Sweet L. J. (2007). Small-molecule ghrelin receptor antagonists improve glucose tolerance, suppress appetite, and promote weight loss. *Endocrinology* 148 5175–5185 17656463 10.1210/en.2007-0239

[B38] Espinosa-de-los-Monteros A. L. Gonzalez B. Vargas G. Sosa E. Mercado M. (2011). Clinical and biochemical characteristics of acromegalic patients with different abnormalities in glucose metabolism. *Pituitary* 14 231–235 21161601 10.1007/s11102-010-0284-x

[B39] Fieffe S. Morange I. Petrossians P. Chanson P. Rohmer V. Cortet C. Borson-Chazot F. Brue T. Delemer B. (2011). Diabetes in acromegaly, prevalence, risk factors, and evolution: data from the French Acromegaly Registry. *Eur. J. Endocrinol.* 164 877–884 21464140 10.1530/EJE-10-1050

[B40] Gahete M. D. Cordoba-Chacon J. Duran-Prado M. Malagon M. M. Martinez-Fuentes A. J. Gracia-Navarro F. Luque R. M. Castano J. P. (2010). Somatostatin and its receptors from fish to mammals. *Ann. N. Y. Acad. Sci.* 1200 43–52 20633132 10.1111/j.1749-6632.2010.05511.x

[B41] Gahete M. D. Duran-Prado M. Luque R. M. Martinez-Fuentes A. J. Vazquez-Martinez R. Malagon M. M. Castano J. P. (2008). Are somatostatin and cortistatin two siblings in regulating endocrine secretions? In vitro work ahead. *Mol. Cell. Endocrinol.* 286 128–134 18215456 10.1016/j.mce.2007.11.013

[B42] Gauna C van der Lely A. J. (2005). Somatostatin, cortistatin, ghrelin and glucose metabolism. *J. Endocrinol. Invest.* 28 127–131 16625861

[B43] Gnanapavan S. Kola B. Bustin S. A. Morris D. G. Mcgee P. Fairclough P. Bhattacharya S. Carpenter R. Grossman A. B. Korbonits M. (2002). The tissue distribution of the mRNA of ghrelin and subtypes of its receptor, GHS-R, in humans. *J. Clin. Endocrinol. Metab.* 87 2988 10.1210/jcem.87.6.8739 12050285

[B44] Golor G. Hu K. Ruffin M. Buchelt A. Bouillaud E. Wang Y. Maldonado M. (2012). A first-in-man study to evaluate the safety, tolerability, and pharmacokinetics of pasireotide (SOM230), a multireceptor-targeted somatostatin analog, in healthy volunteers. *Drug Des. Dev. Ther.* 6 71–79 10.2147/DDDT.S29125 PMC3346155 22573933

[B45] Gomez R. Lago F. Gomez-Reino J. J. Gualillo O. (2009). Novel factors as therapeutic targets to treat diabetes. Focus on leptin and ghrelin. *Expert Opin. Ther. Targets* 13 583–591 19397477 10.1517/14728220902914834

[B46] Gonzalez-Rey E. Chorny A. Delgado M. (2006). Therapeutic action of ghrelin in a mouse model of colitis. *Gastroenterology* 130 1707–1720 16697735 10.1053/j.gastro.2006.01.041

[B47] Gonzalez-Rey E. Delgado M. (2008). Emergence of cortistatin as a new immunomodulatory factor with therapeutic potential in immune disorders. *Mol. Cell. Endocrinol.* 286 135–140 17850953 10.1016/j.mce.2007.08.001

[B48] Granata R. Baragli A. Settanni F. Scarlatti F. Ghigo E. (2010a). Unraveling the role of the ghrelin gene peptides in the endocrine pancreas. *J. Mol. Endocrinol.* 45 107–118 20595321 10.1677/JME-10-0019

[B49] Granata R. Volante M. Settanni F. Gauna C. Ghe C. Annunziata M. Deidda B. Gesmundo I. Abribat T. Van Der Lely A. J. Muccioli G. Ghigo E. Papotti M. (2010b). Unacylated ghrelin and obestatin increase islet cell mass and prevent diabetes in streptozotocin-treated newborn rats. *J. Mol. Endocrinol.* 45 9–17 20382773 10.1677/JME-09-0141

[B50] Granata R. Gallo D. Luque R. M. Baragli A. Scarlatti F. Grande C. Gesmundo I. Cordoba-Chacon J. Bergandi L. Settanni F. Togliatto G. Volante M. Garetto S. Annunziata M. Chanclon B. Gargantini E. Rocchietto S. Matera L. Datta G. Morino M. Brizzi M. F. Ong H. Camussi G. Castano J. P. Papotti M. Ghigo E. (2012a). Obestatin regulates adipocyte function and protects against diet-induced insulin resistance and inflammation. *FASEB J*. 26 3393–3411 22601779 10.1096/fj.11-201343

[B51] Granata R. Settanni F. Julien M. Nano R. Togliatto G. Trombetta A. Gallo D. Piemonti L. Brizzi M. F. Abribat T. Van Der Lely A. J. Ghigo E. (2012b). Des-acyl ghrelin fragments and analogues promote survival of pancreatic beta-cells and human pancreatic islets and prevent diabetes in streptozotocin-treated rats. *J. Med. Chem.* 55 2585–2596 22352743 10.1021/jm201223m

[B52] Granata R. Settanni F. Biancone L. Trovato L. Nano R. Bertuzzi F. Destefanis S. Annunziata M. Martinetti M. Catapano F. Ghe C. Isgaard J. Papotti M. Ghigo E. Muccioli G. (2007). Acylated and unacylated ghrelin promote proliferation and inhibit apoptosis of pancreatic beta-cells and human islets: involvement of 3′,5′-cyclic adenosine monophosphate/protein kinase A, extracellular signal-regulated kinase 1/2, and phosphatidyl inositol 3-kinase/Akt signaling. *Endocrinology* 148 512–529 17068144 10.1210/en.2006-0266

[B53] Granata R. Settanni F. Gallo D. Trovato L. Biancone L. Cantaluppi V. Nano R. Annunziata M. Campiglia P. Arnoletti E. Ghe C. Volante M. Papotti M. Muccioli G. Ghigo E. (2008). Obestatin promotes survival of pancreatic beta-cells and human islets and induces expression of genes involved in the regulation of beta-cell mass and function. *Diabetes* 57 967–979 18162507 10.2337/db07-1104

[B54] Grottoli S. Celleno R. Gasco V. Pivonello R. Caramella D. Barreca A. Ragazzoni F. Pigliaru F. Alberti D. Ferrara R. Angeletti G. (2005). Efficacy and safety of 48 weeks of treatment with octreotide LAR in newly diagnosed acromegalic patients with macroadenomas: an open-label, multicenter, non-comparative study. *J. Endocrinol. Invest.* 28 978–983 16483175 10.1007/BF03345335

[B55] Grottoli S. Gasco V. Broglio F. Baldelli R. Ragazzoni F. Gallenca F. Mainolfi A. Prodam F. Muccioli G. Ghigo E. (2006). Cortistatin-17 and somatostatin-14 display the same effects on growth hormone, prolactin, and insulin secretion in patients with acromegaly or prolactinoma. *J. Clin. Endocrinol. Metab.* 91 1595–1599 16449338 10.1210/jc.2005-1837

[B56] Gualillo O. Lago F. Dieguez C. (2008). Introducing GOAT: a target for obesity and anti-diabetic drugs? Trends Pharmacol. *Sci.* 29 398–401 10.1016/j.tips.2008.06.003 18606462

[B57] Gutierrez J. A. Solenberg P. J. Perkins D. R. Willency J. A. Knierman M. D. Jin Z. Witcher D. R. Luo S. Onyia J. E. Hale J. E. (2008). Ghrelin octanoylation mediated by an orphan lipid transferase. *Proc. Natl. Acad. Sci. U.S.A.* 105 6320–6325 18443287 10.1073/pnas.0800708105 PMC2359796

[B58] Hansen J. B. Arkhammar P. O. Bodvarsdottir T. B. Wahl P. (2004). Inhibition of insulin secretion as a new drug target in the treatment of metabolic disorders. *Curr. Med. Chem.* 11 1595–1615 15180566 10.2174/0929867043365026

[B59] Hansen T. K. Dall R. Hosoda H. Kojima M. Kangawa K. Christiansen J. S. Jorgensen J. O. (2002). Weight loss increases circulating levels of ghrelin in human obesity. *Clin. Endocrinol. (Oxf.)* 56 203–206 11874411 10.1046/j.0300-0664.2001.01456.x

[B60] Hillman J. B. Tong J. Tschop M. (2011). Ghrelin biology and its role in weight-related disorders. *Discov. Med.* 11 521–528 21712018

[B61] Hosoda H. Kojima M. Matsuo H. Kangawa K. (2000). Ghrelin and des-acyl ghrelin: two major forms of rat ghrelin peptide in gastrointestinal tissue. *Biochem. Biophys. Res. Commun.* 279 909–913 11162448 10.1006/bbrc.2000.4039

[B62] Howard A. D. Feighner S. D. Cully D. F. Arena J. P. Liberator P. A. Rosenblum C. I. Hamelin M. Hreniuk D. L. Palyha O. C. Anderson J. Paress P. S. Diaz C. Chou M. Liu K. K. Mckee K. K. Pong S. S. Chaung L. Y. Elbrecht A. Dashkevicz M. Heavens R. Rigby M. Sirinathsinghji D. J. Dean D. C. Melillo D. G. Patchett A. A. Nargund R. Griffin P. R. Demartino J. A. Gupta S. K. Schaeffer J. M. Smith R. G Van Der Ploeg L. H. (1996). A receptor in pituitary and hypothalamus that functions in growth hormone release. *Science* 273 974–977 8688086 10.1126/science.273.5277.974

[B63] Irako T. Akamizu T. Hosoda H. Iwakura H. Ariyasu H. Tojo K. Tajima N. Kangawa K. (2006). Ghrelin prevents development of diabetes at adult age in streptozotocin-treated newborn rats. *Diabetologia* 49 1264–1273 16570155 10.1007/s00125-006-0226-3

[B64] Isgaard J. Granata R. (2011). Ghrelin in cardiovascular disease and atherogenesis. *Mol. Cell. Endocrinol.* 340 59–64 21458527 10.1016/j.mce.2011.03.006

[B65] Jain R. Lammert E. (2009). Cell–cell interactions in the endocrine pancreas. *Diabetes Obes. Metab.* 11(Suppl. 4) 159–167 19817798 10.1111/j.1463-1326.2009.01102.x

[B66] Janson E. T. Oberg K. (1999). Somatostatin receptor ligands and their use in the treatment of endocrine disorders. *Curr. Pharm. Des.* 5 693–705 10495360

[B67] Jawiarczyk A. Bolanowski M. Syrycka J. Bednarek-Tupikowska G. Kaluzny M. Kolodziejczyk A. Domoslawski P. (2012). Effective therapy of insulinoma by using long-acting somatostatin analogue. A case report and literature review. *Exp. Clin. Endocrinol. Diabetes* 120 68–72 22187292 10.1055/s-0031-1287792

[B68] Jeffery P. L. Mcguckin M. A. Linden S. K. (2011). Endocrine impact of *Helicobacter pylori*: focus on ghrelin and ghrelin *o*-acyltransferase. *World J. Gastroenterol.* 17 1249–1260 21455323 10.3748/wjg.v17.i10.1249 PMC3068259

[B69] Kadoglou N. P. Sailer N. Kapelouzou A. Lampropoulos S. Vitta I. Kostakis A. Liapis C. D. (2012). Effects of atorvastatin on apelin, visfatin (nampt), ghrelin and early carotid atherosclerosis in patients with type 2 diabetes. *Acta Diabetol*. 49 269–276 21748474 10.1007/s00592-011-0310-0

[B70] Kageyama H. Funahashi H. Hirayama M. Takenoya F. Kita T. Kato S. Sakurai J. Lee E. Y. Inoue S. Date Y. Nakazato M. Kangawa K. Shioda S. (2005). Morphological analysis of ghrelin and its receptor distribution in the rat pancreas. *Regul. Pept.* 126 67–71 15620416 10.1016/j.regpep.2004.08.031

[B71] Kerem M. Salman B. Ozsoy S. Pasaoglu H. Bedirli A. Haziroglu R. Yilmaz T. U. (2009). Exogenous ghrelin enhances endocrine and exocrine regeneration in pancreatectomized rats. *J. Gastrointest. Surg.* 13 775–783 19082668 10.1007/s11605-008-0778-2

[B72] Kiewiet R. M. van Aken M. O. van der Weerd K. Uitterlinden P. Themmen A. P. Hofland L. J. de Rijke Y. B. Delhanty P. J. Ghigo E. Abribat T van der Lely A. J. (2009). Effects of acute administration of acylated and unacylated ghrelin on glucose and insulin concentrations in morbidly obese subjects without overt diabetes. *Eur. J. Endocrinol.* 161 567–573 19628651 10.1530/EJE-09-0339

[B73] Kineman R. D. Gahete M. D. Luque R. M. (2007). Identification of a mouse ghrelin gene transcript that contains intron 2 and is regulated in the pituitary and hypothalamus in response to metabolic stress. *J. Mol. Endocrinol.* 38 511–521 17496153 10.1677/JME-06-0026

[B74] Kinoshita Y. Fujii H. Takeshita A. Taguchi M. Miyakawa M. Oyama K. Yamada S. Takeuchi Y. (2011). Impaired glucose metabolism in Japanese patients with acromegaly is restored after successful pituitary surgery if pancreatic {beta}-cell function is preserved. *Eur. J. Endocrinol*. 164 467–473 21285083 10.1530/EJE-10-1096

[B75] Kirchner H. Gutierrez J. A. Solenberg P. J. Pfluger P. T. Czyzyk T. A. Willency J. A. Schurmann A. Joost H. G. Jandacek R. J. Hale J. E. Heiman M. L. Tschop M. H. (2009). GOAT links dietary lipids with the endocrine control of energy balance. *Nat. Med.* 15 741–745 19503064 10.1038/nm.1997 PMC2789701

[B76] Kojima M. Hosoda H. Date Y. Nakazato M. Matsuo H. Kangawa K. (1999). Ghrelin is a growth-hormone-releasing acylated peptide from stomach. *Nature* 402 656–660 10604470 10.1038/45230

[B77] Kojima M. Kangawa K. (2005). Ghrelin: structure and function. *Physiol. Rev.* 85 495–522 15788704 10.1152/physrev.00012.2004

[B78] Korbonits M. Grossman A. B. (2004). Ghrelin: update on a novel hormonal system. *Eur. J. Endocrinol.* 151(Suppl. 1) S67–S70 15339247 10.1530/eje.0.151s067

[B79] Kumar R. Salehi A. Rehfeld J. F. Hoglund P. Lindstrom E. Hakanson R. (2010). Proghrelin peptides: desacyl ghrelin is a powerful inhibitor of acylated ghrelin, likely to impair physiological effects of acyl ghrelin but not of obestatin A study of pancreatic polypeptide secretion from mouse islets. *Regul. Pept.* 164 65–70 20619300 10.1016/j.regpep.2010.06.005

[B80] Lear P. V. Iglesias M. J. Feijoo-Bandin S. Rodriguez-Penas D. Mosquera-Leal A. Garcia-Rua V. Gualillo O. Ghe C. Arnoletti E. Muccioli G. Dieguez C. Gonzalez-Juanatey J. R. Lago F. (2010). Des-acyl ghrelin has specific binding sites and different metabolic effects from ghrelin in cardiomyocytes. *Endocrinology* 151 3286–3298 20410201 10.1210/en.2009-1205

[B81] Liu G. Fortin J. P. Beinborn M. Kopin A. S. (2007). Four missense mutations in the ghrelin receptor result in distinct pharmacological abnormalities. *J. Pharmacol. Exp. Ther.* 322 1036–1043 17596538 10.1124/jpet.107.123141

[B82] Liu J. Prudom C. E. Nass R. Pezzoli S. S. Oliveri M. C. Johnson M. L. Veldhuis P. Gordon D. A. Howard A. D. Witcher D. R. Geysen H. M. Gaylinn B. D. Thorner M. O. (2008). Novel ghrelin assays provide evidence for independent regulation of ghrelin acylation and secretion in healthy young men. *J. Clin. Endocrinol. Metab.* 93 1980–1987 18349056 10.1210/jc.2007-2235 PMC2386282

[B83] Liu Y. Zhou Y. B. Zhang G. G. Cai Y. Duan X. H. Teng X. Song J. Q. Shi Y. Tang C. S. Yin X. H. Qi Y. F. (2010). Cortistatin attenuates vascular calcification in rats. *Regul. Pept.* 159 35–43 19766150 10.1016/j.regpep.2009.09.005

[B84] Longo K. A. Charoenthongtrakul S. Giuliana D. J. Govek E. K. Mcdonagh T. Qi Y. Distefano P. S. Geddes B. J. (2008). Improved insulin sensitivity and metabolic flexibility in ghrelin receptor knockout mice. *Regul. Pept.* 150 55–61 18453014 10.1016/j.regpep.2008.03.011

[B85] Lustig R. H. Greenway F. Velasquez-Mieyer P. Heimburger D. Schumacher D. Smith D. Smith W. Soler N. Warsi G. Berg W. Maloney J. Benedetto J. Zhu W. Hohneker J. (2006). A multicenter, randomized, double-blind, placebo-controlled, dose-finding trial of a long-acting formulation of octreotide in promoting weight loss in obese adults with insulin hypersecretion. *Int. J. Obes. (Lond.)* 30 331–341 16158082 10.1038/sj.ijo.0803074 PMC1540404

[B86] Maccario M. Grottoli S. Procopio M. Oleandri S. E. Rossetto R. Gauna C. Arvat E. Ghigo E. (2000). The GH/IGF-I axis in obesity: influence of neuro-endocrine and metabolic factors. *Int. J. Obes. Relat. Metab. Disord.* 24(Suppl. 2) S96–S99 10997620 10.1038/sj.ijo.0801289

[B87] Mazziotti G. Floriani I. Bonadonna S. Torri V. Chanson P. Giustina A. (2009). Effects of somatostatin analogs on glucose homeostasis: a metaanalysis of acromegaly studies. *J. Clin. Endocrinol. Metab.* 94 1500–1508 19208728 10.1210/jc.2008-2332

[B88] Mazziotti G. Gazzaruso C. Giustina A. (2011). Diabetes in Cushing syndrome: basic and clinical aspects. *Trends Endocrinol. Metab.* 22 499–506 21993190 10.1016/j.tem.2011.09.001

[B89] McLaughlin T. Abbasi F. Lamendola C. Frayo R. S. Cummings D. E. (2004). Plasma ghrelin concentrations are decreased in insulin-resistant obese adults relative to equally obese insulin-sensitive controls. *J. Clin. Endocrinol. Metab.* 89 1630–1635 15070922 10.1210/jc.2003-031572

[B90] Moller L. N. Stidsen C. E. Hartmann B. Holst J. J. (2003). Somatostatin receptors. *Biochim. Biophys. Acta* 1616 1–84 14507421 10.1016/s0005-2736(03)00235-9

[B91] Mousseaux D. Le Gallic L. Ryan J. Oiry C. Gagne D. Fehrentz J. A. Galleyrand J. C. Martinez J. (2006). Regulation of ERK1/2 activity by ghrelin-activated growth hormone secretagogue receptor 1A involves a PLC/PKCvarepsilon pathway. *Br. J. Pharmacol.* 148 350–365 16582936 10.1038/sj.bjp.0706727 PMC1751558

[B92] Muccioli G. Baragli A. Granata R. Papotti M. Ghigo E. (2007). Heterogeneity of ghrelin/growth hormone secretagogue receptors. Toward the understanding of the molecular identity of novel ghrelin/GHS receptors. *Neuroendocrinology* 86 147–164 17622734 10.1159/000105141

[B93] Nothacker H. P. Wang Z. Zeng H. Mahata S. K. O’Connor D. T. Civelli O. (2005). Proadrenomedullin N-terminal peptide and cortistatin activation of MrgX2 receptor is based on a common structural motif. *Eur. J. Pharmacol.* 519 191–193 16111673 10.1016/j.ejphar.2005.07.001

[B94] Pantel J. Legendre M. Cabrol S. Hilal L. Hajaji Y. Morisset S. Nivot S. Vie-Luton M. P. Grouselle D. De Kerdanet M. Kadiri A. Epelbaum J. Le Bouc Y. Amselem S. (2006). Loss of constitutive activity of the growth hormone secretagogue receptor in familial short stature. *J. Clin. Invest.* 116 760–768 16511605 10.1172/JCI25303 PMC1386106

[B95] Papotti M. Tarabra E. Allia E. Bozzalla-Cassione F. Broglio F. Deghenghi R. Ghigo E. Muccioli G. (2003). Presence of cortistatin in the human pancreas. *J. Endocrinol. Invest.* 26 RC15–RC18 14669820 10.1007/BF03347348

[B96] Patel Y. C. (1999). Somatostatin and its receptor family. *Front. Neuroendocrinol.* 20 157–198 10433861 10.1006/frne.1999.0183

[B97] Petersenn S. Schopohl J. Barkan A. Mohideen P. Colao A. Abs R. Buchelt A. Ho Y. Y. Hu K. Farrall A. J. Melmed S. Biller B. M. (2010). Pasireotide (SOM230) demonstrates efficacy and safety in patients with acromegaly: a randomized, multicenter, phase II trial. *J. Clin. Endocrinol. Metab.* 95 2781–2789 20410233 10.1210/jc.2009-2272

[B98] Poykko S. M. Kellokoski E. Horkko S. Kauma H. Kesaniemi Y. A. Ukkola O. (2003). Low plasma ghrelin is associated with insulin resistance, hypertension, and the prevalence of type 2 diabetes. *Diabetes* 52 2546–2553 14514639 10.2337/diabetes.52.10.2546

[B99] Prado C. L. Pugh-Bernard A. E. Elghazi L. Sosa-Pineda B. Sussel L. (2004). Ghrelin cells replace insulin-producing beta cells in two mouse models of pancreas development. *Proc. Natl. Acad. Sci. U.S.A.* 101 2924–2929 14970313 10.1073/pnas.0308604100 PMC365721

[B100] Qader S. S. Hakanson R. Rehfeld J. F. Lundquist I. Salehi A. (2008). Proghrelin-derived peptides influence the secretion of insulin, glucagon, pancreatic polypeptide and somatostatin: a study on isolated islets from mouse and rat pancreas. *Regul. Pept*. 146 230–237 17942170 10.1016/j.regpep.2007.09.017

[B101] Qader S. S. Lundquist I. Ekelund M. Hakanson R. Salehi A. (2005). Ghrelin activates neuronal constitutive nitric oxide synthase in pancreatic islet cells while inhibiting insulin release and stimulating glucagon release. *Regul. Pept*. 128 51–56 15721487 10.1016/j.regpep.2004.12.018

[B102] Qi Y. Longo K. A. Giuliana D. J. Gagne S. Mcdonagh T. Govek E. Nolan A. Zou C. Morgan K. Hixon J. Saunders J. O. Distefano P. S. Geddes B. J. (2011). Characterization of the insulin sensitivity of ghrelin receptor KO mice using glycemic clamps. *BMC Physiol.* 11 1 10.1186/1472-6793-11-1 PMC3024223 21211044

[B103] Regard J. B. Sato I. T. Coughlin S. R. (2008). Anatomical profiling of G protein-coupled receptor expression. *Cell* 135 561–571 18984166 10.1016/j.cell.2008.08.040 PMC2590943

[B104] Reinehr T. De Sousa G. Roth C. L. (2008). Obestatin and ghrelin levels in obese children and adolescents before and after reduction of overweight. *Clin. Endocrinol. (Oxf.)* 68 304–310 17854392 10.1111/j.1365-2265.2007.03042.x

[B105] Ren A. J. Guo Z. F. Wang Y. K. Lin L. Zheng X. Yuan W. J. (2009). Obestatin, obesity and diabetes. *Peptides* 30 439–444 18992781 10.1016/j.peptides.2008.10.002

[B106] Resmini E. Minuto F. Colao A. Ferone D. (2009). Secondary diabetes associated with principal endocrinopathies: the impact of new treatment modalities. *Acta Diabetol.* 46 85–95 19322513 10.1007/s00592-009-0112-9

[B107] Saad M. F. Bernaba B. Hwu C. M. Jinagouda S. Fahmi S. Kogosov E. Boyadjian R. (2002). Insulin regulates plasma ghrelin concentration. *J. Clin. Endocrinol. Metab.* 87 3997–4000 12161550 10.1210/jcem.87.8.8879

[B108] Sahin I. Aydin S. Ozkan Y. Dagli A. F. Akin K. O. Guzel S. P. Catak Z. Ozercan M. R. (2011). Diet-induced obesity suppresses ghrelin in rat gastrointestinal tract and serum. *Mol. Cell. Biochem.* 355 299–308 21556824 10.1007/s11010-011-0867-5

[B109] Sakata I. Yang J. Lee C. E. Osborne-Lawrence S. Rovinsky S. A. Elmquist J. K. Zigman J. M. (2009). Colocalization of ghrelin *O*-acyltransferase and ghrelin in gastric mucosal cells. *Am. J. Physiol. Endocrinol. Metab.* 297 E134–E141 19401456 10.1152/ajpendo.90859.2008 PMC2711663

[B110] Saltiel A. R. Kahn C. R. (2001). Insulin signalling and the regulation of glucose and lipid metabolism. *Nature* 414 799–806 11742412 10.1038/414799a

[B111] Sato T. Nakamura Y. Shiimura Y. Ohgusu H. Kangawa K. Kojima M. (2012). Structure, regulation and function of ghrelin. *J. Biochem.* 151 119–128 22041973 10.1093/jb/mvr134

[B112] Schellekens H. Dinan T. G. Cryan J. F. (2010). Lean mean fat reducing “ghrelin” machine: hypothalamic ghrelin and ghrelin receptors as therapeutic targets in obesity. *Neuropharmacology* 58 2–16 19573543 10.1016/j.neuropharm.2009.06.024

[B113] Seim I. Amorim L. Walpole C. Carter S. Chopin L. K. Herington A. C. (2010). Ghrelin gene-related peptides: multifunctional endocrine/autocrine modulators in health and disease. *Clin. Exp. Pharmacol. Physiol.* 37 125–131 19566830 10.1111/j.1440-1681.2009.05241.x

[B114] Seim I. Collet C. Herington A. C. Chopin L. K. (2007). Revised genomic structure of the human ghrelin gene and identification of novel exons, alternative splice variants and natural antisense transcripts. *BMC Genomics* 8 298 10.1186/1471-2164-8-298 PMC2014779 17727735

[B115] Shenouda M. Maldonado M. Wang Y. Bouillaud E. Hudson M. Nesheiwat D. Hu K. (2012). An open-label dose-escalation study of once-daily and twice-daily pasireotide in healthy volunteers: safety, tolerability, and effects on glucose, insulin, and glucagon levels. *Am. J. Ther.* 10.1097/MJT.0b013e31824c3eb4 [Epub ahead of print]. 22713526

[B116] Solomon T. P. Chambers E. S. Jeukendrup A. E. Toogood A. A. Blannin A. K. (2008). The effect of feeding frequency on insulin and ghrelin responses in human subjects. *Br. J. Nutr.* 100 810–819 18394217 10.1017/S000711450896757X

[B117] Souza-Moreira L. Campos-Salinas J. Caro M. Gonzalez-Rey E. (2011). Neuropeptides as pleiotropic modulators of the immune response. *Neuroendocrinology* 94 89–100 21734355 10.1159/000328636

[B118] Strowski M. Z. Blake A. D. (2008). Function and expression of somatostatin receptors of the endocrine pancreas. *Mol. Cell. Endocrinol.* 286 169–179 18375050 10.1016/j.mce.2008.02.007

[B119] Sun Y. Asnicar M. Saha P. K. Chan L. Smith R. G. (2006). Ablation of ghrelin improves the diabetic but not obese phenotype of ob/ob mice. *Cell Metab*. 3 379–386 16679295 10.1016/j.cmet.2006.04.004

[B120] Sun Y. Garcia J. M. Smith R. G. (2007). Ghrelin and growth hormone secretagogue receptor expression in mice during aging. *Endocrinology* 148 1323–1329 17158206 10.1210/en.2006-0782

[B121] Togliatto G. Trombetta A. Dentelli P. Baragli A. Rosso A. Granata R. Ghigo D. Pegoraro L. Ghigo E. Brizzi M. F. (2010). Unacylated ghrelin rescues endothelial progenitor cell function in individuals with type 2 diabetes. *Diabetes* 59 1016–1025 20068135 10.2337/db09-0858 PMC2844809

[B122] Tong J. Prigeon R. L. Davis H. W. Bidlingmaier M. Kahn S. E. Cummings D. E. Tschop M. H D’Alessio D. (2010). Ghrelin suppresses glucose-stimulated insulin secretion and deteriorates glucose tolerance in healthy humans. *Diabetes* 59 2145–2151 20584998 10.2337/db10-0504 PMC2927935

[B123] Toshinai K. Yamaguchi H. Sun Y. Smith R. G. Yamanaka A. Sakurai T. Date Y. Mondal M. S. Shimbara T. Kawagoe T. Murakami N. Miyazato M. Kangawa K. Nakazato M. (2006). Des-acyl ghrelin induces food intake by a mechanism independent of the growth hormone secretagogue receptor. *Endocrinology* 147 2306–2314 16484324 10.1210/en.2005-1357

[B124] Tostivint H. Lihrmann I. Bucharles C. Vieau D. Coulouarn Y. Fournier A. Conlon J. M. Vaudry H. (1996). Occurrence of two somatostatin variants in the frog brain: characterization of the cDNAs, distribution of the mRNAs, and receptor-binding affinities of the peptides. *Proc. Natl. Acad. Sci. U.S.A.* 93 12605–12610 8901629 10.1073/pnas.93.22.12605 PMC38039

[B125] Tschop M. Smiley D. L. Heiman M. L. (2000). Ghrelin induces adiposity in rodents. *Nature* 407 908–913 11057670 10.1038/35038090

[B126] Tschop M. Weyer C. Tataranni P. A. Devanarayan V. Ravussin E. Heiman M. L. (2001). Circulating ghrelin levels are decreased in human obesity. *Diabetes* 50 707–709 11289032 10.2337/diabetes.50.4.707

[B127] Tzotzas T. Papazisis K. Perros P. Krassas G. E. (2008). Use of somatostatin analogues in obesity. *Drugs* 68 1963–1973 18778119 10.2165/00003495-200868140-00003

[B128] Ueberberg B. Unger N. Saeger W. Mann K. Petersenn S. (2009). Expression of ghrelin and its receptor in human tissues. *Horm. Metab. Res.* 41 814–821 19670151 10.1055/s-0029-1233462

[B129] Ukkola O. (2011). Genetic variants of ghrelin in metabolic disorders. *Peptides* 32 2319–2322 21527298 10.1016/j.peptides.2011.04.013

[B130] van der Hoek J. van der Lelij A. J. Feelders R. A. De Herder W. W. Uitterlinden P. Poon K. W. Boerlin V. Lewis I. Krahnke T. Hofland L. J. Lamberts S. W. (2005). The somatostatin analogue SOM230, compared with octreotide, induces differential effects in several metabolic pathways in acromegalic patients. *Clin. Endocrinol. (Oxf.)* 63 176–184 16060911 10.1111/j.1365-2265.2005.02322.x

[B131] van der Lely A. J. Tschop M. Heiman M. L. Ghigo E. (2004). Biological, physiological, pathophysiological, and pharmacological aspects of ghrelin. *Endocr. Rev.* 25 426–457 15180951 10.1210/er.2002-0029

[B132] Velasquez-Mieyer P. A. Umpierrez G. E. Lustig R. H. Cashion A. K. Cowan P. A. Christensen M. Spencer K. A. Burghen G. A. (2004). Race affects insulin and GLP-1 secretion and response to a long-acting somatostatin analogue in obese adults. *Int. J. Obes. Relat. Metab. Disord.* 28 330–333 14708034 10.1038/sj.ijo.0802561 PMC1490020

[B133] Veldhuis J. D. Bowers C. Y. (2010). Integrating GHS into the Ghrelin System. *Int. J. Pept.* 2010 879503 10.1155/2010/879503 PMC2925380 20798846

[B134] Venables M. C. Jeukendrup A. E. (2009). Physical inactivity and obesity: links with insulin resistance and type 2 diabetes mellitus. *Diabetes Metab. Res. Rev.* 25(Suppl. 1) S18–S23 19662619 10.1002/dmrr.983

[B135] Verhulst P. J. Depoortere I. (2012). Ghrelin's second life: from appetite stimulator to glucose regulator. *World J. Gastroenterol*. 18 3183–3195 22783041 10.3748/wjg.v18.i25.3183 PMC3391754

[B136] Vestergaard E. T. Gormsen L. C. Jessen N. Lund S. Hansen T. K. Moller N. Jorgensen J. O. (2008). Ghrelin infusion in humans induces acute insulin resistance and lipolysis independent of growth hormone signaling. *Diabetes* 57 3205–3210 18776138 10.2337/db08-0025 PMC2584125

[B137] Volante M. Allia E. Gugliotta P. Funaro A. Broglio F. Deghenghi R. Muccioli G. Ghigo E. Papotti M. (2002). Expression of ghrelin and of the GH secretagogue receptor by pancreatic islet cells and related endocrine tumors. *J. Clin. Endocrinol. Metab.* 87 1300–1308 11889202 10.1210/jcem.87.3.8279

[B138] Wierup N. Svensson H. Mulder H. Sundler F. (2002). The ghrelin cell: a novel developmentally regulated islet cell in the human pancreas. *Regul. Pept*. 107 63–69 12137967 10.1016/s0167-0115(02)00067-8

[B139] Wierup N. Yang S. Mcevilly R. J. Mulder H. Sundler F. (2004). Ghrelin is expressed in a novel endocrine cell type in developing rat islets and inhibits insulin secretion from INS-1 (832/13) cells. *J. Histochem. Cytochem.* 52 301–310 14966197 10.1177/002215540405200301

[B140] Wortley K. E. Del Rincon J. P. Murray J. D. Garcia K. Iida K. Thorner M. O. Sleeman M. W. (2005). Absence of ghrelin protects against early-onset obesity. *J. Clin. Invest.* 115 3573–3578 16322795 10.1172/JCI26003 PMC1297252

[B141] Wren A. M. Small C. J. Abbott C. R. Dhillo W. S. Seal L. J. Cohen M. A. Batterham R. L. Taheri S. Stanley S. A. Ghatei M. A. Bloom S. R. (2001). Ghrelin causes hyperphagia and obesity in rats. *Diabetes* 50 2540–2547 11679432 10.2337/diabetes.50.11.2540

[B142] Wren A. M. Small C. J. Ward H. L. Murphy K. G. Dakin C. L. Taheri S. Kennedy A. R. Roberts G. H. Morgan D. G. Ghatei M. A. Bloom S. R. (2000). The novel hypothalamic peptide ghrelin stimulates food intake and growth hormone secretion. *Endocrinology* 141 4325–4328 11089570 10.1210/endo.141.11.7873

[B143] Yang J. Zhao T. J. Goldstein J. L. Brown M. S. (2008). Inhibition of ghrelin *O*-acyltransferase (GOAT) by octanoylated pentapeptides. *Proc. Natl. Acad. Sci. U.S.A.* 105 10750–10755 18669668 10.1073/pnas.0805353105 PMC2504781

[B144] Yoshimoto A. Mori K. Sugawara A. Mukoyama M. Yahata K. Suganami T. Takaya K. Hosoda H. Kojima M. Kangawa K. Nakao K. (2002). Plasma ghrelin and desacyl ghrelin concentrations in renal failure. *J. Am. Soc. Nephrol.* 13 2748–2752 12397045 10.1097/01.asn.0000032420.12455.74

[B145] Zhu X. Cao Y. Voogd K. Steiner D. F. (2006). On the processing of proghrelin to ghrelin. *J. Biol. Chem.* 281 38867–38870 17050541 10.1074/jbc.M607955200

[B146] Zigman J. M. Nakano Y. Coppari R. Balthasar N. Marcus J. N. Lee C. E. Jones J. E. Deysher A. E. Waxman A. R. White R. D. Williams T. D. Lachey J. L. Seeley R. J. Lowell B. B. Elmquist J. K. (2005). Mice lacking ghrelin receptors resist the development of diet-induced obesity. *J. Clin. Invest.* 115 3564–3572 16322794 10.1172/JCI26002 PMC1297251

